# Suitability of potyviral recombinant virus-like particles bearing a complete food allergen for immunotherapy vaccines

**DOI:** 10.3389/fimmu.2022.986823

**Published:** 2022-09-08

**Authors:** Diego Pazos-Castro, Clémence Margain, Zulema Gonzalez-Klein, Marina Amores-Borge, Carmen Yuste-Calvo, Maria Garrido-Arandia, Lucía Zurita, Vanesa Esteban, Jaime Tome-Amat, Araceli Diaz-Perales, Fernando Ponz

**Affiliations:** ^1^ Centre for Plant Biotechnology and Genomics, Universidad Politécnica de Madrid – Instituto Nacional de Investigación y Tecnología Agraria y Alimentaria / Consejo Superior de Investigaciones Científicas (UPM–INIA/CSIC), Universidad Politécnica de Madrid, Madrid, Spain; ^2^ Department of Biotechnology-Plant Biology, Escuela Técnica Superior de Ingeniería Agronómica, Alimentaria y de Biosistemas (ETSIAAB), Universidad Politécnica de Madrid, Madrid, Spain; ^3^ Department of Allergy and Immunology, Instituto de Investigación Sanitaria (IIS)-Fundación Jiménez Díaz, Universidad Autónoma de Madrid (UAM), Madrid, Spain

**Keywords:** virus-like particles, antigen delivery, food allergy, immunotherapy, plant biotechnology, turnip mosaic virus, Pru p 3

## Abstract

Virus-like particles (VLPs) have been gaining attention as potential platforms for delivery of cargos in nanomedicine. Although animal viruses are largely selected due to their immunostimulatory capacities, VLPs from plant viruses constitute a promising alternative to be considered. VLPs derived from Turnip mosaic virus (TuMV) have proven to present a tridimensional structure suited to display molecules of interest on their surface, making them interesting tools to be studied in theragnostic strategies. Here, we study their potential in the treatment of food allergy by genetically coupling TuMV-derived VLPs to Pru p 3, one of the most dominant allergens in Mediterranean climates. VLPs-Pru p 3 were generated by cloning a synthetic gene encoding the TuMV coat protein and Pru p 3, separated by a linker, into a transient high-expression vector, followed by agroinfiltration in *Nicotiana benthamiana* plants. The generated fusion protein self-assembled *in planta* to form the VLPs, which were purified by exclusion chromatography. Their elongated morphology was confirmed by electron microscopy and their size (~400 nm), and monodispersity was confirmed by dynamic light scattering. Initial *in vitro* characterization confirmed that they were able to induce proliferation of human immune cells. This proliferative capability was enhanced when coupled with the natural lipid ligand of Pru p 3. The resultant formulation, called VLP-Complex, was also able to be transported by intestinal epithelial cells, without affecting the monolayer integrity. In light of all these results, VLP-Complex was furtherly tested in a mouse model of food allergy. Sublingual administration of VLP-Complex could effectively reduce some serological markers associated with allergic responses in mice, such as anti-Pru p 3 sIgE and sIgG2a. Noteworthy, no associated macroscopic, nephritic, or hepatic toxicity was detected, as assessed by weight, blood urea nitrogen (BUN) and galectin-3 analyses, respectively. Our results highlight the standardized production of allergen-coated TuMV-VLPs in *N. benthamiana* plants. The resulting formula exerts notable immunomodulatory properties without the need for potentially hazardous adjuvants. Accordingly, no detectable toxicity associated to their administration was detected. As a result, we propose them as good candidates to be furtherly studied in the treatment of immune-based pathologies.

## Graphical Abstract

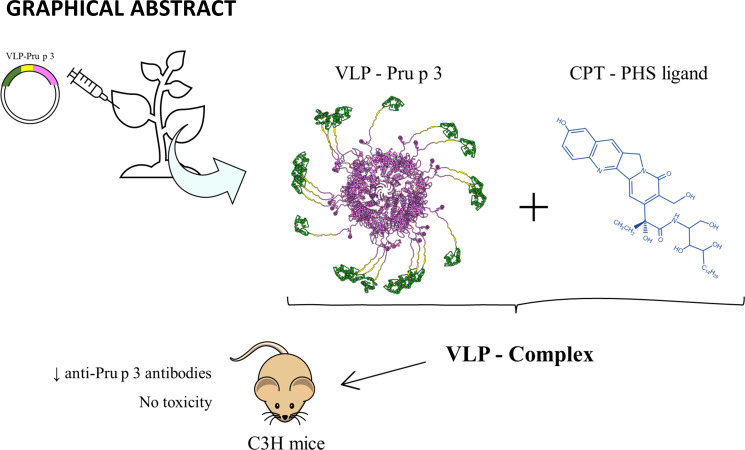

## Introduction

In nanobiotechnology, virus-like particles (VLPs) are increasingly becoming an important set of nanoparticles useful for a high number of applications. VLPs are very similar to virions but lack the corresponding encapsidated nucleic acid. Globally, both types of particles are usually referred to as viral nanoparticles (VNPs). Plant-derived VLPs in particular are the subject of important developments in several areas (1). Within plant VLPs, particles with a high aspect ratio can be found, both rigid and flexuous ones. Rod-type particles derived from tobacco mosaic virus (rigid) or potato virus X (flexuous) have received most of the attention in this context (2), but VLPs derived from turnip mosaic virus (TuMV), a flexuous rod-type potyvirus, have also shown an important potential for nanobiotechnological applications. Thus, chemically or genetically functionalized TuMV VLPs have been exploited for applications as diverse as antibody sensing, enzyme nano-immobilization, antimicrobials, or biofabrication (3).

In nanomedicine, VLPs have been widely used as pharmacological formulations against Alzheimer’s (4), arthritis (5), atherosclerosis (6) and cancer (7–9) as well as infections, such as malaria, papillomavirus, and the ongoing SARS-CoV-2 pandemic, among others (10, 11). VLPs can be used as delivery platforms to transport cargos inside their structures specifically to the desired target, since their self-assembly capacity can be tightly controlled to adopt several architectures of interest (12–14). In addition, the viral coat can be attached to the cargo by both chemical fusion or genetic engineering, resulting in the production of vehicles displaying multiple subunits of the active component on their surface (15). As a result, higher effective doses of the compound are concentrated in a small area, enabling the activation of the immune system without the need of potentially toxic adjuvants with undesirable side effects, although adjuvants could also be included in the formulation if needed (16, 17). In addition, nanostructures with highly repeated domains are well-known activators of the human immune system, since epitopes displayed in an organized repetitive form induce strong activation of the B cell repertoire (18–20), as well as dendritic cells (21).

Food allergy has been increasing in prevalence over the past decades, with an estimation of around 5% of the global population suffering from it, doubling this figure when focusing on infants (22). The World Health Organization and the European Academy of Allergy and Clinical Immunology have calculated the very high economical costs derived from the prevention and treatment of allergies, and even higher when related to unexpected reactions due to hidden allergens (23). Thus, regardless of the obvious advances in immunotherapy treatments (24), the general recommendation from clinicians is still to avoid the contact with the allergen sources (25). The molecules in the food responsible for allergy triggering in susceptible individuals are the allergens, most of which are proteins and/or lipoproteins (26–28). Over the last few years, increasing attention is being paid to the application of nanotechnology (nanoparticles) to specific applications such as allergen detection, diagnosis and allergen-specific immunotherapy (AIT) (29–32).

**Figure 1:**
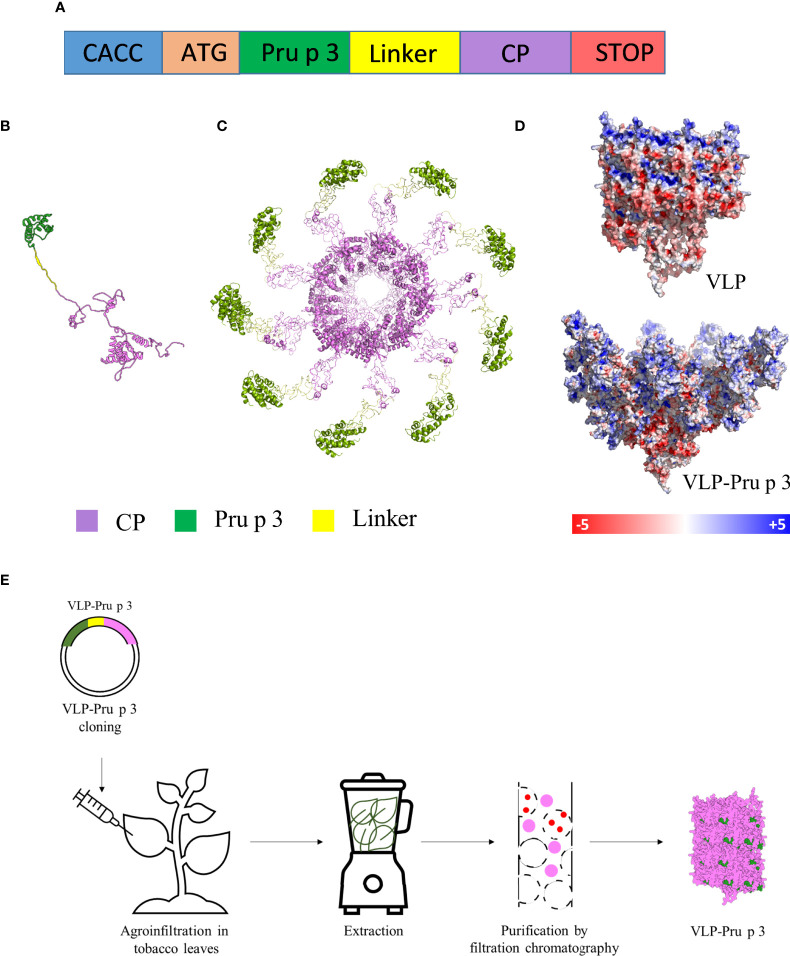
Production of VLP-Pru p 3. **(A)** Schematic representation of the synthetic genetic constructs coding for the recombinant fusion proteins to be expressed in plants. CACC sequence for directional cloning into the Gateway Entry vector. ATG initiation codon. Pru p 3 peach allergen sequence. Linker sequence to provide physical separation of Pru p 3 and CP. Two different linkers were tried. CP TuMV coat protein sequence. STOP codon. The relative lengths of the different modules in the constructs are not represented at scale. **(B)** Tertiary structure of the CP-Pru p 3 subunit as modelled by I-TASSER. **(C)** Quaternary structure of VLP-Pru p 3. **(D)** Poisson-Boltzmann electrostatic potential mapped onto the outer surface of VLP and VLP-Pru p 3. **(E)** Schematic diagram of VLP-Pru p 3 production and purification.

AIT is the only treatment that can eradicate the allergic phenotype in a patient. In contrast, its use is not extended through food allergenic patients since better benefit/risk ratios need to be reached before introducing them in clinical routine practices (25, 33, 34). One of the main disadvantages these formulations face nowadays is the fact that AITs are usually produced using extracts purified from the allergenic source, which makes it difficult to standardize the actual amount of allergen administered to the patient. Besides, the presence of multiple allergens in extracts cannot be discarded, increasing the probability of undesired cross-reactivity and side effects (35, 36). In this work, we introduce the design, standardized production, and characterization of an allergen-coated TuMV-based VLP (**Figure 1**), and we study its potential application as a novel AIT formulation in food allergy management, taking Pru p 3 as a model, one of the most characterized food allergens from peach (27, 37, 38).

Pru p 3 is a 9 kDa basic protein belonging to the Lipid Transfer Protein (LTP) family of allergens. This family is characterized by the presence of a hydrophobic tunnel in the protein capable of binding several lipids *in vitro*, although in nature these proteins are always found with the same ligand (39, 40). Their folded structure presents four disulphide bridges that confers the protein high thermal stability and pH resistance, being able to maintain its functionality at high temperatures and extreme pH (both basic and acid) (28, 39). Pru p 3 is the major allergen from peach (41). However, patients sensitized to it can also develop allergic reactions when consuming other fruits from the *Rosaceae* family, especially apple, apricot and plum (42). Clinically, allergic reactions to LTPs can include a variety of symptoms, ranging from moderate manifestations (urticaria, oral allergy syndrome, vomiting…) to life-threatening anaphylactic responses (27). All these symptoms appear as consequence of the existence of specific anti-LTP IgE (sIgE) in the patients sera (43), which gets attached to high affinity receptors (FcƐRI) present in mast cell and basophils’ surface. Cross-linking of IgE and cognate allergens (in this case, LTPs) in the surface of effector cells leads to the release of proinflammatory mediators (histamine, leukotrienes, prostaglandins…) that are responsible for the clinical symptoms previously described (44). For this reason, measurement of sIgE levels in sera constitutes a routine method commonly used by physicians to diagnose LTP allergy (27). In addition, other immunoglobulin subtypes, such as sIgG1 and sIgG2a, can also be usually identified in murine models of the disease (41).

Pru p 3 is found in nature in complex with its ligand, an alkaloid derivative bound to phytosphingosine, also known as camptothecin-phytosphingosine (CPT-PHS) ligand (39, 40). Previous works have shown the importance of the carried ligands in the sensitization phase of allergy development, both to LTPs and other proteins (45, 46). The deep characterization of Pru p 3 mechanism of action using *in vitro*, *in vivo*, and *in silico* approaches has not only helped us to understand the mechanisms underlying food allergy sensitization, but also to use Pru p 3 as a model to study LTP allergy (45, 47). Thus, Pru p 3 is an excellent allergen of reference for nanobiotechnological developments based on the use of VLPs. We have approached such developments through the exposure of Pru p 3 on the external surface of the TuMV VLP *via* genetic fusion, linked by a linker. The recombinant protein has been expressed in plants to prompt self-assembly within the agroinfiltrated plant cells. The formed VLPs have been characterized and their potential both for sensing allergen-specific antibodies and AIT have been explored.

## Results

### Construction, production, and purification of recombinant CP-Pru p 3

The synthetic gene construct to be expressed in plants for TuMV coat protein (CP) fused to Pru p 3 (CP-Pru p3) (**Figure 1A**) was cloned in a pEAQ expression vector, and agroinfiltrated in *N. benthamiana* plants using *Agrobacterium tumefaciens*, in parallel with the construct expressing the unmodified TuMV CP for its use as control. In order to analyze the stability of the structure and the possible physicochemical consequences of fusing Pru p 3 to it, an in silico modelling was performed. The initial structure obtained was minimized by means of 10 ns molecular dynamics simulations (**Figure 1B**). As shown in **Figure 1C**, Pru p 3 is arranged on the TuMV surface of the nanoparticles and produce a change in the molecular surface of the virion, but also in the distribution of the electrostatic potential (**Figure 1D**). The presence of Pru p 3 produces an increase in the positive electrostatic regions of the VLPs surface that could modify the protein-protein interactions exerted by the nanoparticle.

**Figure 2:**
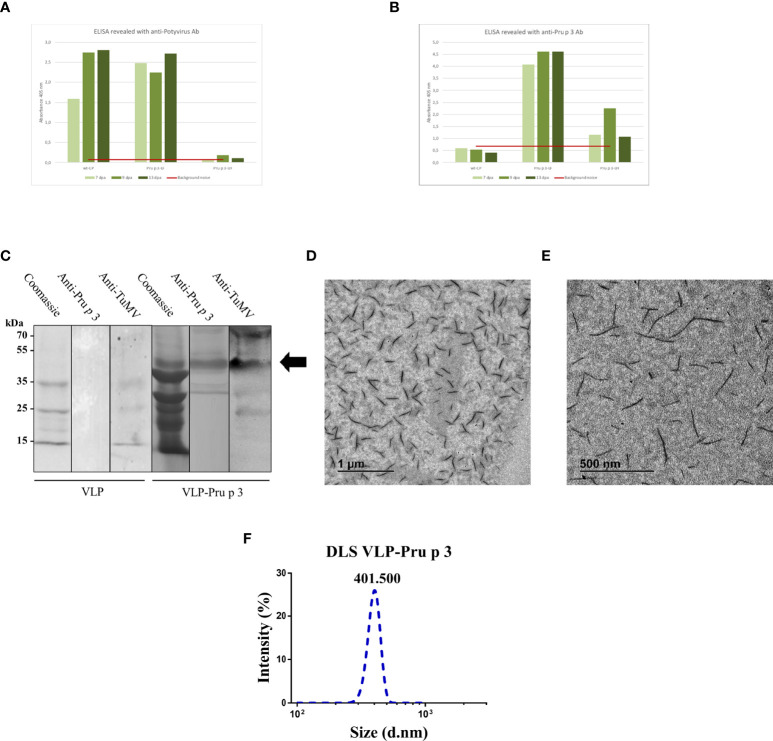
Characterization of VLP-Pru p 3 production. ELISA analyses of extracts from *Nicotiana benthamiana* leaves, using **(A)** anti-Potyvirus, or **(B)** anti-Pru p 3 antibodies. Readings at 405 nm are shown of leaves collected at 7, 9, or 13 days post-agroinfiltration (dpa). Red line (background noise) was calculated as the three-fold absorbance of non-infiltrated leaves. **(C)** Western blot analyses from exclusion chromatography fractions from VLP and VLP-Pru p 3 agroinfiltrates. **(D, E)** Representative transmission electron microscope images of VLP-Pru p 3. **(F)** Representative analysis of VLP-Pru p 3 size and dispersion by DLS. The assay was repeated weekly for 6 consecutive weeks to assess stability of the formulation through time.

Two weeks after agroinfiltration, leaves were collected and frozen at -80 °C until use. VLPs were purified as described in the *Methods* section (**Figure 1E**). Two different linkers were tried (flexible and helicoidal), aimed to provide physical separation between Pru p 3 and the CP, and different results were obtained with each of them. In fact, the aspect of the leaves infiltrated with the different constructs suggested that their expressions and productions in the plant tissue were quite different (**Supplementary Figure 1**). ELISA analyses showed that the different linkers tested had a relevant effect on the expression levels of the CP-Pru p 3 protein (**Figures 2A, B**). Thus, absorbance readings with the monoclonal antibody against potyvirus CP, were clearly positive for the wild-type CP construct and the construct incorporating the flexible linker (LF) at any time analyzed, but just at, or barely over, background levels for the helicoidal linker (LH) (**Figure 2A**). When the polyclonal anti-Pru p 3 was used, the constructs with both linkers were positive, but the flexible one gave much higher readings (**Figure 2B**). In light of these results, the CP-Pru p 3 with flexible linker was the chosen construct to be characterized in the following assays.

### VLP-Pru p 3 characterization

After purification, CP-Pru p 3 yields were 10-30 mg/100 g of agroinfiltrated leaves. SDS-PAGE and immunoblotting analysis of the produced VLP-Prup3 showed the presence of a protein with lower mobility than the wild-type CP, and compatible with the theoretical molecular weight, with positive signal when blotted against anti-TuMV and anti-Pru p 3 antibodies (**Figure 2C**).

Assembly of CP-Pru p 3 subunits into VLP-Pru p 3 was assessed by transmission electron microscopy, which confirmed the presence of VLPs in the preparations (**Figures 2D, E**). Particles were quite homogeneous (monodisperse), although shorter (~400 nm) than the typical length (~720 nm) of TuMV virions, wild-type CP VLPs, or other TuMV-based recombinant constructs (48–50). Dynamic light scattering (DLS) analysis confirmed these results (**Figure 2F**), as well as the homogeneity of the VLP population (average polydispersity index > 0.9).

### Complex displayed in VLP scaffolds increases its immunostimulatory capacity *in vitro*

**Figure 3:**
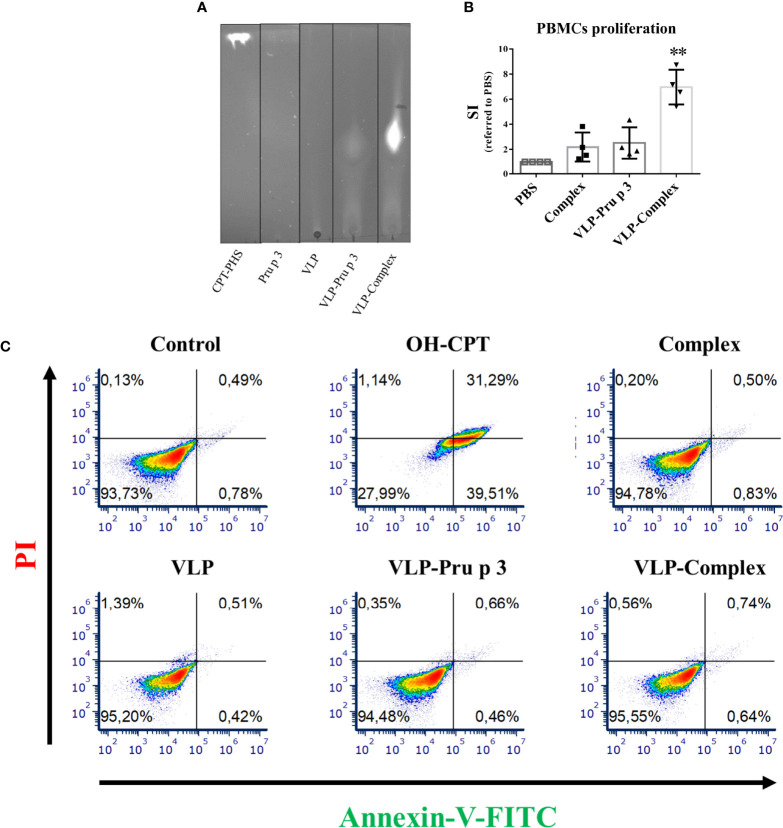
*In vitro* effects of VLP-Complex over immune cells. **(A)** Assessment of VLP-Complex formation by TLC. Lipidic fraction was detected by emission under UV light, due to CPT activity. **(B)** Proliferation assay of human PBMCs from healthy volunteers (n = 4), stimulated with Complex, VLP-Pru p 3 or VLP-Complex. After 5 days, cell numbers were determined by flow cytometry and SI was calculated as described in Methods. Data are presented as mean (SD, Kruskal-Wallis test with Dunn’s correction for multiple comparisons). **P < 0.01. **(C)** Effects of VLP, VLP-Pru p 3 and VLP-Complex on monocyte apoptosis by Annexin V-PI detection. To quantify the toxicity of the production, they were incubated with THP1 (cells of monocyte origin) for 16 hours. After that, annexin V or PI were added to quantify cell mortality. Four technical replicates were performed for each stimulus. Representative results are shown.

In order to assess the correct folding of Pru p 3 in the VLP assemblies, VLP-Pru p 3 were incubated with CPT-PHS ligand, which was chemically synthetized as previously described (40), thus forming VLP-Complex. After dialyzing to ensure the removal of ligand excess, VLP-Complex was analyzed by thin layer chromatography (TLC), which confirmed that the complex had been formed (**Figure 3A**).

Previous reports indicate that complex formation is vital to induce an immunological response against Pru p 3, using both *in vitro* and *in vivo* approaches. Accordingly, complex formation guarantees NF-κB activation in monocytes (40) and promotes allergic sensitization in experimental mice more efficiently than Pru p 3 alone (46). In addition, stimulation of human peripheral blood mononuclear cells (PBMCs) with complex induces stronger proliferation ratios when compared to non-complexed Pru p 3 stimulation (45). To assess if these results could be reproduced with VLP-based formulations, PBMCs from healthy volunteers were cultured with VLP-Pru p 3 and VLP-Complex. As shown in **Figure 3B**, complex induced a two-fold proliferation of PBMCs when referred to unstimulated controls, similar to previous reports (45). Whereas VLP-Pru p 3 also induced the same SI (stimulation index), VLP-Complex induced a much stronger proliferation ratio (seven-fold SI when referred to controls). This result suggests that the display of complex in the surface of VLP scaffolds enhances its immunostimulatory capacity. Noteworthy, this was not accompanied by an increase in its immunotoxicity, since monocytes cultured with VLP-Complex did not incorporate neither annexin V nor propidium iodide (PI), as assessed by flow cytometry (**Figure 3C**). This suggests they did not induce neither apoptosis nor necrosis in the studied cell line in those conditions. Altogether, these results make the TuMV VLP-Complex a good candidate to be studied in-depth for its possible application in drug designing for immune-based pathologies.

### VLPs-Complex are transported by Caco-2 cells without affecting epithelial integrity

**Figure 4:**
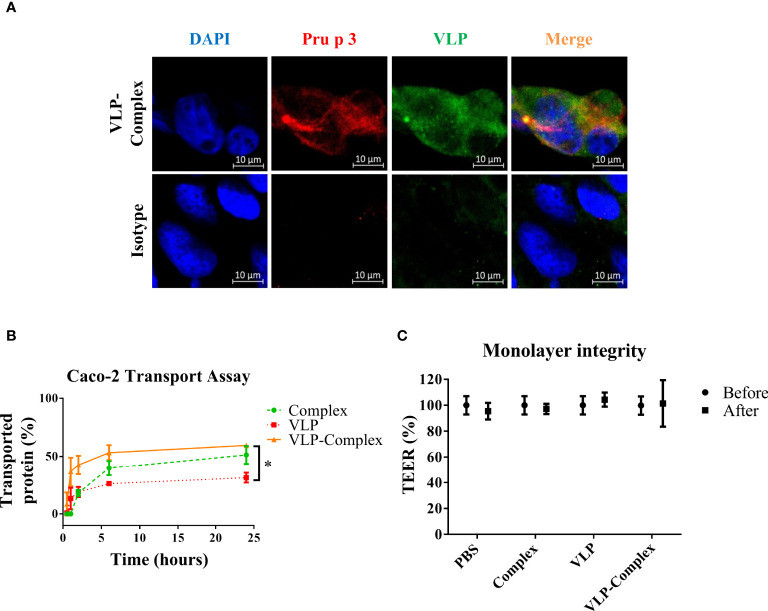
*In vitro* effects of VLP-Complex on epithelial cells. **(A)** Detection of VLP-Complex inside Caco-2 cells by immunofluorescence. Blue: DAPI, red: Pru p 3, green: VLP. Bar = 10 µm. **(B)** Detection by ELISA of VLP and VLP-Complex transported through a monolater of Caco-2 cells (in Transwell™ format), using anti-VLP antibodies. *P < 0.05. **(C)** Monolayer integrity assessment by TEER measurement. All assays were performed in four technical replicates. Data are presented as mean (SD, Mann-Whitney test).

To use VLP-Complex in nanotherapeutic formulations, their ability to be assimilated and transported by human epithelia must be first studied. As shown in **Figure 4A**, VLP-Complex was detected inside Caco-2 cells 2 h after stimulus addition, by immunofluorescence and confocal microscopy. To assess if a Caco-2-based epithelium could transport the formulation from an apical to a basolateral side, these cells were grown in Transwell^®^ format as previously described (51). Complex, VLP and VLP-Complex were added to the apical side of the monolayer, and transport ratios of these formulations to the basolateral side were quantified by ELISA. 24 h after being added to the apical side, ~50% of complex had been transported through the epithelial barrier, which was similar to the percentage of VLP-Complex transported in the same period (~60%; **Figure 4B**). Transport of VLP-Complex was significantly greater than that of VLP alone (~30%). Noteworthy, in all cases transepithelial electrical resistance (TEER) values remained unaffected 24 h after stimulus addition (**Figure 4C**).

### VLPs-Complex alter the serological immune profiling of allergic mice

**Figure 5:**
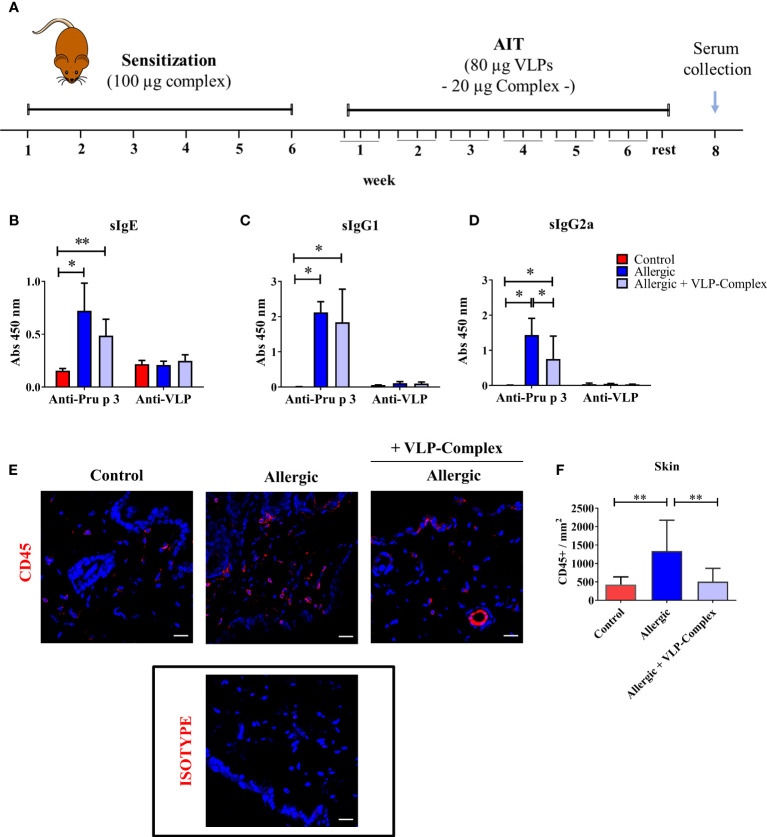
*In vivo* effectivity of VLP-Complex. **(A)** Schematic diagram showing the experimental procedures followed to sensitize and treat C3H mice (Control n = 5; Allergic n = 5; Allergic + VLP-Complex n = 9). Six consecutive weeks of sensitization were followed by six consecutive weeks of VLP-Complex sublingual treatment. After resting for one week, mice were euthanized by CO_2_ suffocation and blood was collected by cardiac puncture. Levels of antigen specific (Pru p 3 or VLP) **(B)** sIgE, **(C)** sIgG1, or **(D)** sIgG2a were assessed by ELISA. Each mouse was analyzed in triplicate. Data are presented as mean (SD, Mann-Whitney test). *P < 0.05, **P < 0.01. **(E)** Representative images and **(F)** quantification of skins and hybridized with anti-CD45 antibody (red). Nuclei (blue) stained with DAPI. 546-labelled anti-goat IgG was used as an isotype control. Quantification of CD45+ cell infiltration was calculated as the number of CD45+ per mm2 (n = 5/group; at least 3 sections were separately stained from each mouse at distal depths of the tissue and 3-5 images were taken per section). Data are presented as mean (SD. Mann-Whitney test). **P<0.01. Bar = 20 µm.

Once the immunological activity and safety of VLP-Complex were shown *in vitro*, we sought to characterize these parameters *in vivo*. We chose Pru p 3 allergy as a model of inflammatory pathology and we developed a mouse model of the disease, based on previously published reports (45, 46). After sensitization, allergic mice received VLP-Complex sublingually as AIT, thrice per week for six consecutive weeks (**Figure 5A**). One week after the last AIT administration, mice were euthanized and levels of serological anti-Pru p 3 antibodies were measured as indicators of the allergic state of the mice (45). As expected, allergic mice developed significant high levels of anti-Pru p 3 sIgE, sIgG1 and sIgG2a antibodies, (**Figure 5B–D**). VLP-Complex administration altered this serological profile, by significantly reducing sIgG2a levels in allergic specimens in almost a two-fold ratio (**Figure 5D**). We also observed a downward tendency in sIgE levels (P = 0.06), the most well-known biomarker associated with allergic symptomatology (52) (**Figure 5B**), although anti-Pru p 3 sIgG1 remained unaltered (**Figure 5C**).

In addition, mice sensitized epicutaneously to Pru p 3 are known to present significantly greater levels of CD45+ cells in this organ than naïve controls (46). However, after sublingual treatment with VLP-Complex, CD45+ infiltration was effectively reverted towards control levels, as assessed by immunofluorescence and confocal microscopy as previously described (46) (**Figures 5E, F**). This result suggests that sublingual administration of VLP-Complex can drive changes in the immune populations of peripheral organs, such as the skin. Nonetheless, more in-depth studies should be conducted in the future to determine which immune populations are being affected and how this affects the global immunological state of the allergic specimen.

### VLP-Complex did not induce any detectable signs of toxicity in mice

**Figure 6:**
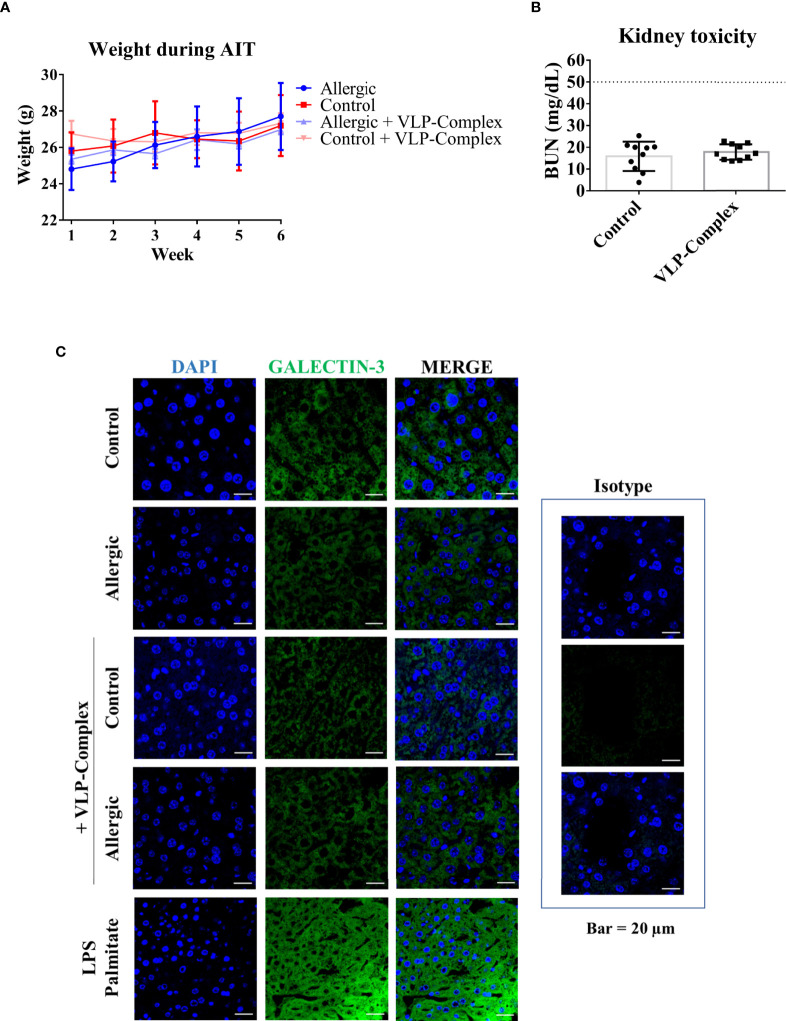
*In vivo* toxicity of VLP-Complex. **(A)** Weight evolution of mice during the six consecutive weeks receiving VLP-Complex (Allergic n = 4; Allergic + VLP-Complex n = 8; Control n = 4; Control + VLP-Complex n = 10). **(B)** Determination of blood urea nitrogen levels from Control (n = 10) and VLP-Complex (n = 10) mice. Each mouse was analyzed in triplicates. Data are presented as mean (SD, Mann-Whitney test). **(C)** Detection of galectin-3 in paraffined livers from Control (n = 3), Allergic (n = 3), Control + VLP-Complex (n = 3) and Allergic + VLP-Complex (n = 3) mice. As a positive control of hepatic stress, livers from control specimens were treated for 72 h with LPS (1 ng/µL) + palmitate (100 ng/µL), following paraffinization and immunofluorescence as described in *Methods*. Five images were taken from each mouse. Representative images are shown. Blue: DAPI, green: galectin-3. Bar = 20 µm.

As well known, safety is a critical point to check in drug development. Therefore, we performed several studies to analyzed VLP-Complex toxicity. Remarkably, sublingually administered VLP-Complex did not induce production of antibodies targeting the CP itself (**Figures 5B–D**). This result might be an indicator of the innocuity of the VLP platform in this context, that would act as an effective carrier for the presented molecule, but without exerting notable adverse effects *in vivo*. In accordance with this observation, there was no reduction in the weight of mice receiving VLP-Complex (**Figure 6A**), as an indicative of their good health status over the procedure. Besides, levels of BUN (blood urea nitrogen) also remained unaltered after VLP-Complex administration, and in all cases were lower than the established threshold in impaired renal function conditions (50 mg/dL) (53), thus denoting no associated kidney toxicity (**Figure 6B**). Finally, detection of galectin-3 in liver was performed, although no changes in its expression and intracellular distribution could be observed by means of immunofluorescence and confocal microscopy (**Figure 6C**), denoting no associated liver toxicity (54).

## Discussion

The way allergens are formulated in AIT-based vaccines has proven to determine the efficacy obtained with these formulations (55). Although it is desirable to present allergens in a context that favors anti-allergic responses, such as in VLP formulations, sometimes the complexity of the resultant constructs forces researchers to change the allergen structure to include only one or two IgE-binding regions, rather than the complete allergen itself. This might compromise the efficacy of some formulas, since they are skewed to the considered epitope in each case (56). The work described in this paper deals with the production of TuMV-based VLPs displaying a complete food allergen (Pru p 3) on its surface, as well as its validation as a formula to successfully induce the presentation of the allergen to the organism.

The production of the VLP-Pru p 3 formula by agroinfiltration of *N. benthamiana* plants proved to be successful. VLP formation was confirmed by both transmission electron microscopy and DLS approaches. Particles were proved to be monodisperse, most of them with a relatively small size in the range of ~400 nm, which is slightly smaller than that previously described for the virus (720 nm) (3). Reduction in VLP size when compared to original virions has been previously described in the literature for rod-shaped constructions. For example, while Papaya Mosaic Virus (PapMV) virions have a canonical length of 500 nm (57), Denis et al. reported that their size is sharply reduced to 150 nm (30% of their original length) in absence of genetic material (58). This length was furtherly decreased to 70 nm and 100 nm after being coupled to influenza’s M2e and HA11 peptides, respectively (58, 59), suggesting that final size is also affected by the antigen being coupled. Despite this shift in length, the PapMV-M2e VLPs displayed notable protective effects against influenza infection in mice, including stimulation of the antibody response and an increase of 50% in the survival rate of the infected mice (58). Thus, at least for plant viruses, it seems unlikely that a reduction in VLP size can alter the downstream application they are designed for, although this must be studied on a case-by-case basis. For TuMV-derived VLP-Complex, the size reduction did not prevent the formulation to induce the serologic conversion of the treated mice, as shown in **Figures 5B–D**. Also, it should be borne in mind that the fact that Pru p 3 coupling could affect VLP size was not completely unexpected, given the large size of the polypeptide (~90 residues) when compared to other antigens that had been previously used in the past with these VLPs, all of them less than 20 residues long (49, 50).

TuMV-derived nanoparticles genetically functionalized with Pru p 3 could be obtained for their characterization and further applications without many complications. This is a relatively remarkable result, considering the functionalization site in the viral CP, and the structural characteristics of the allergen. The insertion of the synthetic Pru p 3 gene was at the CP N-terminal domain, within an amino acid stretch which could not be structurally solved by cryoelectronic microscopy (60). This is usually due to a high degree of flexibility and disorder in the domain which, in the case of viral proteins, has been often related to assembly requirements (61). Pru p 3 crystal structure (60) revealed a highly ordered globular conformation with four disulphide bridges, which is a quite different structural situation. Our previous experience with genetically fused structured small proteins or peptides had shown the incapability to assemble VLPs (49, 62), but this was not the case for Pru p 3, a protein larger than others previously tried. We still do not have an obvious explanation for this discrepancy, although the fact that Pru p 3 was physically separated from the CP by a linker most likely contributed to the alleviation of possible structural tensions. The differential results obtained with the two linkers assayed tend to reinforce this view, since the most structured one, the helical, performed notably worse than the flexible one.

In addition to the correct coupling of the VLP assemblies, it was confirmed that Pru p 3 was as well adequately folded. As a member of the LTP family, the 3D structure of Pru p 3 includes a hydrophobic tunnel which accommodates lipids (28, 40). The correct formation of this tunnel is vital for the protein to establish a stable interaction with its natural CPT-PHS ligand, with a dissociation constant in the range of µM (40). Our results show that VLP-Pru p 3 particles are able to bind the CPT-PHS ligand (thus forming VLP-Complex), which suggests that the tunnel is correctly formed and that Pru p 3 presents an LTP-characteristic topology. This is crucial to guarantee that the allergen will be exposed to the immune system in a similar fashion as it is found naturally in the food, preserving its IgE-binding regions and its immunogenic activity (63). In order to guarantee that VLPs would have access to the immune system, we first studied their ability to be transported by epithelial tissues. Due to their size, we initially hypothesized that VLPs would be poorly transported. However, our results with Caco-2 monolayers show that up to 25% of the VLPs can cross the barrier after 6 h of incubation with the cells. This relatively high transport ratio of the nanoparticles might be explained due to their filamentous morphology, as opposed to icosahedral viruses. Rod-shaped particles display very low diameters (13-15 nm) (64) despite their large length (in our case, 400 nm), which might facilitate their transport by the paracellular route, rather than by transcytosis (65). In fact, rod-shaped VLPs have previously shown some advantages over icosahedral structures, such as greater accessibility to tumor environments in anti-cancer treatments (2). It should be remarked that these high transport ratios of VLP-based formulas were obtained without a significant decrease in TEER values after VLP addition. This observation shows that: a) VLP-based formulations, at the concentrations studied, exert no toxicity over epithelial cells, so they constitute safe candidates to be used in nanotherapeutic formulations; and b) the obtained transport ratios are not an artifact due to epithelial impairment. Altogether, these results confirmed that VLPs can cross epithelial barriers and that the allergen delivered on their surface is correctly folded. One limitation of this study is that we have not been able to determine which receptor mediates the entry of TuMV-derived VLPs in Caco-2 cells. In fact, we do not know if this interaction is indeed established through a specific surface molecule (such as in the case of cowpea mosaic virus and vimentin), or if TuMV particles are able to display nonspecific cell entry, as it happens with many wild-type plant viruses (1). Nonetheless, determining this would be interesting to fine tune VLP biodistribution *in vivo*.

We next sought to evaluate the immunogenicity of the formula by incubation of human PBMCs with VLP-Pru p 3. Under these conditions, PBMCs exerted a proliferation ratio increased by 2-fold when compared to unstimulated controls. Since Pru p 3 has been described to induce stronger proliferative responses when coupled to its lipid ligand (45, 46), we sought to test if the addition of CPT-PHS (i.e., VLP-Complex) could enhance the aforementioned proliferative response. Indeed, this new formulation exhibited an enhanced proliferation, increased by 3-fold when compared to VLP-Pru p 3 alone. This result may be explained due to the ligand activity itself (40, 45, 46), for example, due to its immunostimulatory properties when it is phosphorylated by human enzymes (40).

Based on the satisfactory results derived from the *in vitro* assays, a pilot preclinical testing was launched to study the use of VLP-Complex as an AIT for food allergy treatment in a food allergic mouse model. Since the decrease in allergen specific IgE levels has been proposed as a surrogate marker for AIT effectivity (66), we measured the levels of this biomarker in the blood of allergic mice receiving the formulation. We observed a downward tendency in specific-Pru p 3 IgE in these mice, suggesting an initial remission of the allergic phenotype, which was confirmed as statistically significant in the case of the sIgG2a isotype. This supports the election of VLP-Complex as a promising candidate to be studied in-depth to treat LTP allergy. However, levels of anti-Pru p 3 sIgG1 remained unaltered after VLP-Complex treatment. It has been reported that most of the high affinity sIgE-secreting plasma cells derive from sIgG1+ memory B cells, which undergo class-switch recombination after exposure to the cognate allergen (67, 68). In contrast, sIgE+ memory B cells in peripheral blood from allergic patients are scarce (69). Thus, the persistently high sIgG1 levels after VLP-Complex administration might be suggesting an important limitation of the current formula, i.e., its inability to efficiently remove long-lasting memory sIgG1+ memory B cells from the organism. Nonetheless, more studies must be conducted before making any assumptions about how VLP-Complex administration affects the B cell compartment *in vivo*. Understanding that interaction is currently a priority objective, as it will help to refine the formulation to obtain full serological reconversion in the treated mice.

Other viruses have been used as scaffolds to develop AIT formulations, as it has been recently reviewed by Bachmann et al. (55). Cucumber mosaic virus (CuMV) VLPs have been modified to include an epitope derived from tetanus toxin (TT), making the resultant CuMVtt more immunogenic (4). When functionalized with cat or peanut allergens, this platform has proven to be a successful tool to alleviate allergic reactions to these compounds in mouse models of the disease (4, 70). The authors suggest that the mechanism of action of this therapy relies on the generation of protective IgG antibodies that antagonize the perilous effects of elevated IgE in the organism (70). In a similar fashion, Potato virus Y VLPs have been coupled to cat allergens to generate an AIT formulation that favors an increase in allergen-specific protective antibodies in healthy experimental mice (71).

In contraposition, our results have been obtained without the use of adjuvants or modifications in the TuMV coat sequence to enhance its immunogenicity (such as the TT epitope), which could trigger undesirable side effects, thus reducing patient adherence to treatment (16). In fact, early toxicity studies suggest that, although they are highly immunogenic *in vitro*, VLPs-Complex do not induce cell death and can pass through the epithelial monolayer without damaging it. Besides, as shown in **Figure 4B**, after 6 h of incubation with intestinal epithelial cells, 50% of VLP-Complex is secreted to the basolateral side. Thus, half-life of the formula in the epithelial mucosa is very short. In this line, monocytes cultured with the formulation stained negative with annexin V and PI, as assessed by flow cytometry analyses. When a cell enters in apoptosis, it starts to express phosphatidylserine in the external domain of its cellular membrane (72). Annexin V has well-known affinity for this phospholipid and, thus, detection of surface annexin V by flow cytometry has been extensively used as a surrogate marker for apoptosis (73). On the other hand, PI has affinity for nucleic acids and, thus, it can bind to nucleic DNA. However, under steady conditions PI cannot cross the cellular membrane and, thus, living cells do not incorporate it. Only when the cell has entered into late apoptotic responses, also known as necrosis, PI gets access to the nucleus and attaches to the DNA. Thus, detection of PI by flow cytometry is used as a surrogate marker for necrotic responses (74). In our case, none of these compounds attached to monocytes after treatment with VLPs or VLP-derived formulations, which might be an indicative of their innocuity in these cells, at least for the concentration studied. In this line, when administered sublingually to mice, no liver damage or kidney toxicity was detected, which suggests that accumulation in these organs, if present, is minimal. This is supported by the fact that well-known nephro- and hepatotoxic drugs, such as cisplatin and paracetamol respectively, induce an increase of BUN and galectin-3 levels as fast as 72 h post-administration in rodents (75, 76). However, none of these markers were elevated in mice treated with VLP-Complex under our dosage and posology.

On the other hand, the mechanism of action of VLP-Complex seems to differ from the one proposed for other AIT formulations. In our case, this mechanism does not seem to rely on the production of protective antibodies, but rather on the re-education of the immune system that prompts a reduction in allergen specific IgE and IgG2a levels in the organism. There is not enough data yet to confirm if the differences observed between the formulas rely on the VLPs themselves or are dependent on the route of administration chosen. Anti-peanut CuMVtt and anti-cat Potato virus Y VLPs were administered subcutaneously to the mice (70, 71). Nonetheless, our results were obtained with a sublingual administration, a route that has shown good adherence ratios in patients with respiratory allergy (77).

In any case, it should be borne in mind that the molecular mechanism by which VLP-Complex interacts with the immune system has not been fully described yet. Generally, the immunological characteristics of VLPs are dependent on repetitive and particular structures and the induction of innate immunity through the activation of pathogen-associated molecular pattern recognition receptors (78). In addition, it has been described that biomolecules present in physiological fluids (such as plasma) can adsorb to the surface of VLPs, altering their biochemical properties and modifying the way in which they interact with the organism. This phenomenon, called protein corona, is dependent on each VLP intrinsic characteristics and, thus, it should be studied individually to determine how it affects each VLP-based formulation that has been described up to now. For example, differences in surface charge could explain alterations in the serological profile induced by the formulations (79).

Nonetheless, the immunological mechanisms behind the serological reconversion observed in mice, as well as studies about the long-term effects of tolerance maintenance, should be conducted and discussed in future publications. We cannot discard that, regarding results derived from those studies, changes in dosage and posology of the formulation could be applied in order to optimize it. Addition of adjuvants in the future to overcome possible limitations cannot be discarded, either. However, the results described here justify the launch of larger scale preclinical models that might give answers to these questions.

## Conclusions

In vaccine manufacture, production platforms must be simple. Here we demonstrate that Pru p 3 can be genetically fused to the CP of TuMV produced in plants. In summary, our results show that TuMV-based VLPs have proven to be a promising antigen-presenting platform. Specifically, our VLP-Complex formulation has exerted immunoregulatory properties, both *in vitro* and *in vivo*, without showing associated immune, hepatic, or nephritic toxicity. Given that its low-scale production has been standardized, and that there is an urgent need to improve existing AIT formulations to make its translation to clinical routine feasible, we believe that these VLP-Complex constitute a promising candidate to be furtherly studied. In addition, its production in *N. benthamiana* plants makes their production relatively easy to scale up, thus guaranteeing their large-scale production through molecular farming strategies.

## Methods

### Pru p 3 cloning in the expression vector. Plant growing and agroinfiltration

A synthetic gene containing the cDNA sequence of Pru p 3 fused to the CP gene of TuMV was ordered from GeneArt (ThermoFisher Scientific) and cloned in the pEAQ-HT-DEST1 expression vector (80). Both sequences (Pru p 3 and CP) were connected by one of two linkers (flexible or helicoidal) designed to provide physical separation between both parts of the fusion protein.

The pEAQ constructs were expressed in plants of *Nicotiana benthamiana* by agroinfiltration of the corresponding *Agrobacterium tumefaciens* (LB 4404 strain), for the production of the TuMV-derived VLPs (49).

### Protein production, purification and assembly verification

Agroinfiltrated leaves of *Nicotiana benthamiana* were recollected and crashed in 0.25M potassium phosphate buffer, pH 7.5, 0.5 M NaCl. The extract was mixed thoroughly with chloroform and after centrifugation 10 minutes at 3500 rpm, the aqueous phase was dialyzed in 0.5M ammonium acetate buffer. VLPs were purified using filtration chromatography (Sephacryl S-200, GE Healthcare; 0.5M NH_4_ Acetate buffer). The production of the fusion protein (CP-Pru p 3) or TuMV wild-type CP was assessed by ELISA, SDS-PAGE and Western blot. All these quality controls of expression and production were carried out as previously described (49, 81). For the techniques involving the use of antibodies (ELISA, Western blot) the identity of the protein expressed was assessed using antibodies against Potyvirus (SRA 27200; Agdia) and antibodies produced against Pru p 3 (51).

VLP assembly was assessed in plant extracts as described previously (50). Grids were examined on a transmission electron microscopy (JEM JEOL 1010, Tokyo, Japan) in an external service (TEM, ICTS-CNME, Madrid, Spain). Samples were immunodecorated with a polyclonal anti-Pru p 3 antibody. Additionally, VLP-Pru p 3 assemblies were analyzed with a Zetasizer Nano ZS by DLS (82). Dispersant was ultrapure water with a temperature of 25°C, a viscosity of 0.8872 mPa·s and a refractive index of 1.33. Sample temperature was set at 25 °C and equilibrium time was set at 120 s. Measurements were performed using Malvern’s DTS0012 disposable cuvettes, at three different angles (13°, 90° and 173°). 5 consecutive measurements were made for each sample, in triplicates. Results were analyzed with ZS XPLORER 2.2.0.147 software and graphed with GraphPad6 (GraphPad Software Inc., La Jolla, CA, USA).

### Structure modelling

CP-Pru p 3 subunit was modelled by protein threading using the I-TASSER server (https://zhanggroup.org/I-TASSER/) (83–85). Quaternary structure of VLP-Pru p 3 assemblies was obtained by structural superposition of modelled CP-Pru p 3 subunits using Chimera software (http://www.rbvi.ucsf.edu/chimera) (86). TuMV (PDB entry 6T34) was used as a scaffold (64). System was minimized by means of Molecular Dynamics simulations using the CHARMM 3.1 force field and the multicore CUDA version of NAMD 2.13 in the Tesla V100 GPU of the high-performance computing CBGP in an isothermal-isobaric ensemble. It was immersed in periodic rectangular solvation boxes with a spacing distance of 20 Å and water molecules added according TIP3P model. Ions were added providing 0.150 M salt concentration. Optimization was performed along 5000 minimization steps, followed by equilibration of water for 100 ps at 2 fs time steps at 298 K and 1 atm with all atoms, except those of water (fixed for 50000 steps). Lastly, simulation ran during 10 ns. Results were processed and analyzed with VMD 1.9.3 and molecular graphics were prepared and rendered with Pymol 2.3.2.

### *In vitro* stimulation of immune cells by VLP-based formulations

To determine if VLP-based therapies can induce immune cell proliferation, PBMCs from different non-allergic volunteers were isolated using a Lymphoprep density gradient. PBMCs were then seeded in flat-bottom 96-well plates at a concentration of 2·10^5^ cells/mL and incubated with VLP-Pru p 3 or VLP-Complex (20 ng/µL VLP; 5 ng/µL Pru p 3 or complex). Complex (5 ng/µL) was used as a positive control to induce proliferation. After 5 days (37°C, 5% CO_2_), cellular concentration was calculated using a BD Accuri cytometer. SI for each stimulus was calculated as the variation of cell concentration over time normalized with the variation of the control.

To evaluate the immunotoxicity of the formulations, human monocytes (THP1 cells; *In vivo*gen) were seeded in flat-bottom 96-well plates at a concentration of 1·10^6^ cells/mL and incubated with the same stimuli as described above. 10-hidroxycamptothecin (0.25 ng/µL) was used as a positive control to induce apoptosis. After 24 h, cells were stained with annexin V-FITC (1:100, Merck) and PI (1:100, Merck) for 10 min (RT). Samples were analyzed using a BD Accuri cytometer and results were processed using FCS Express 7 Plus software. Unstained samples were used as background controls.

### Immunolocalization of VLP-Complex in intestinal epithelial cells

Caco-2 cells (ATCC HTB-37; human intestinal epithelium) were seeded into poly-L-lysine-treated, 2 cm^2^ round coverslips (ThermoFisher), at a concentration of 2·10^5^ cells/cover in 250 µL of supplemented DMEM. After 48 h of growth (37°C, 5% CO_2_), VLP-Complex was added (36 ng/µL VLP; 9 ng/µL complex) and incubated for 2 hours at 37°C. After extensive washing with PBS, cells were fixed with 4% formaldehyde (ThermoFisher) for 10 min (RT). Nuclei were stained by treatment with PBS 0.1% Triton 0.72 mM DAPI for 7 min and blocking was performed for 45 min (RT) with 1% BSA. Samples were then incubated with anti-TuMV antibodies (CAB 18700; Agdia) and anti-Pru p 3, for 1h (RT). Finally, specimens were mounted with ProLong Gold Antifade Mountant (ThermoFisher). Images were obtained with a Zeiss LSM 880 confocal microscope, using 405 and 561 nm laser excitations. Graphical material was analyzed with ZEN 3.1 software.

### Transport assay

Transwell™ plates (24-wells) were seeded with Caco-2 cells as previously described (51). TEER was measured to assess monolayer integrity, before and one week after stimuli addition. VLP or VLP-Complex (20 ng/µL VLP; 5 ng/µL Complex) were added to the apical side of the monolayer, and FBS-free basolateral media were collected at different time points. After being dialyzed in 0.1 M ammonium acetate O/N (4°C) using Spectra/Por^®^ 3.5 kDa membranes (Spectrum Labs), samples were freeze-dried and resuspended in equal volumes of PBS. Samples were then analyzed by ELISA, by coating 96-well polystyrene microtiter plaques (Corning^®^ Costar, Merck) with the resuspension O/N (4°C). Subsequently, wells were blocked with Casein Blocking Buffer (Merck) for 1 h (RT) and incubated with anti-TuMV antibody for another hour (RT), followed by extensive washing with PBS and 1 h incubation with anti-rabbit-HRP. Signal was developed with 1-Step Ultra TMB ELISA (ThermoFisher) and the reaction was stopped with 2N HCl. Absorbance (450 nm) was measured with a SPECTROstar Nano microplate reader (BMG LABTECH).

### Animals

Female specific pathogen free C3H mice (6-to-8-week-old) were purchased from Charles River (L’Arbresle, France). All animals were randomly assigned and separated into each group immediately after its arrival to our facilities. They were fed *ad libitum* with a Pru p 3-free diet (Labdiet Eurodent Diet 22% pellet for rodents). All the procedures were carried at the IIS- Fundación Jiménez Díaz (FJD, Madrid, Spain).

### *In vivo* Pru p 3 allergy model

Mice were sensitized epicutaneously with complex [Pru p 3 and its associated lipid ligand (40)], based on previously published reports (45, 46). Briefly, abdominal fur was removed (once per week, for 6 consecutive weeks) by application of depilatory cream. Immediately after each depilation, mice were anesthetized with inhaled isoflurane and placed in supine position. Complex (100 µg in 50 µL PBS) were added in the depilated area, until dry (~45-60 min). Age- and sex-matched depilated, non-sensitized animals were used as controls.

### *In vivo* VLP-Complex administration

Posology of VLP-Complex consisted in 3 weekly doses of 80 µg VLP (20 µg complex), for 6 consecutive weeks. Briefly, allergic and control mice were anesthetized by ketamine:xylazine intraperitoneal injection and placed in supine position. Therapy was administered sublingually, in 20 µL 0.5M ammonium acetate. Age- and sex-matched allergic animals received 20 µL 0.5M ammonium acetate as control treatment. Weight was recorded at the beginning of each week. One week after the last VLP-Complex administration, mice were euthanized by CO_2_ inhalation. Blood samples were taken post-mortem by cardiac punction, and sera was isolated by centrifugation (10 min, 3000 g). For immunofluorescence analyses, liver biopsies were collected and included in paraffin.

### Serologic profiling

Sera were analyzed by ELISA, to detect both anti-TuMV or anti-Pru p 3 specific antibodies. For sIgE detection, 384-well ELISA plates were coated with VLPs/Pru p 3 (5 µg/mL) for 2 h at 37°C, followed by blocking (1h, RT) with 1% BSA. Sera (1:4 dilution) were incubated O/N at 4°C, followed by extensive washing and incubation with anti-IgE-HRP antibody (1:2000; PA1-84764; Invitrogen). For sIgG1 and sIgG2a evaluation, 96-well ELISA plates were coated with the same antigens and blocked with Casein Blocking Buffer (Merck) for 1 h (RT). Sera (1:25 dilution) were incubated O/N at 4°C, followed by extensive washing and incubation with anti-IgG1 (1:5000; A90-105A; Bethyl) or anti-IgG2a (1:5000; A90-107A; Bethyl) antibodies. After washing, incubation with anti-goat-HRP antibody was performed. For all cases, signal was developed as described in the *Transport assay* section.

In addition, BUN was measured as a biomarker for nephrotoxicity, using the Urea Nitrogen Colorimetric Detection Kit (Invitrogen), following provider’s instructions. For this analysis, sera were used at a 1:20 dilution. Absorbance (450 nm) was measured with a SPECTROstar Nano microplate reader (BMG LABTECH).

### Hepatotoxicity assessment by galectin-3 detection in liver

Paraffined liver biopsies (7 µm sections) were cut using a microtome (Leica). After deparaffination and rehydration, specimens underwent antigen retrieval by heating at 87°C for 10 min in 0.12% Tris 0.037% EDTA 0.05% Tween (pH 9.0). After 15 min of cool-down at RT, samples were washed, and nuclei were stained with PBS 0.1% Triton 0.72 mM DAPI for 7 min. Blocking was performed for 1 h (RT) with 10% BSA, and anti-galectin-3 antibody (1:100; SAB4501746-100UG; Merck) was added for 1 h (RT). After extensive washing with PBS, secondary anti-rabbit-546 antibody was added for 1h (RT). Finally, specimens were mounted with ProLong Gold Antifade Mountant (ThermoFisher). Images were obtained with a Zeiss LSM 880 confocal microscope, using 405 and 561 nm laser excitations. Graphical material was analyzed with ZEN 3.1 software.

### Statistical analyses

Statistically significant differences were assessed by GraphPad6 using Mann-Whitney test, except where noted. In all cases, P values < 0.05 were considered significant.

## Data availability statement

The original contributions presented in the study are included in the article/**Supplementary Material**. Further inquiries can be directed to the corresponding authors.

## Ethics statement

All human volunteers were included in the research after providing informed consent. All experimental protocols were conducted in accordance with the latest revision of the Declaration of Helsinki after being approved by the ethical committee from Universidad Politécnica de Madrid (LILIPAL_BIO2017-84548-R). The patients/participants provided their written informed consent to participate in this study. All animal experiments were performed with the permission of the Institutional Animal Care and Use Committee from the Community of Madrid (Ref. PROEX 074.4/21), under the current legislation (European Union Directive 2010/63/EU) and in compliance to ARRIVE guidelines.

## Author contributions

DP-C: Conceptualization, investigation, writing original draft. CM: Investigation. ZG-K: Conceptualization, investigation, writing review. MA-B: Investigation. CY-C: Investigation. MG-A: Investigation, writing review. LZ: Investigation. VE: Writing review, resources, funding acquisition. JT-A: Conceptualization, investigation, writing original draft. AD-P: Conceptualization, writing original draft, resources, funding acquisition. FP: Conceptualization, writing review, resources, funding acquisition. All authors contributed to the article and approved the submitted version.

## Funding

This research was funded by the Community of Madrid through the project FOODAL (FOODAL-CM; S2018/BAA-4574) co-funded by ESF and ERDF R&D projects call Tecnologías 2018. Work was also supported by the Spanish Ministry of Science and Innovation through the project LISENTRA, granted by the Spanish Research State Agency (PID2020-113629RB00/AEI/10.13039/501100011033), and by Instituto de Salud Carlos III co-funded by ERDF RETIC program ARADyAL (RD16/0006/0003). DP-C was granted by Universidad Politécnica de Madrid and Banco Santander for a predoctoral Programa Propio grant. ZG-K, CY-C and JT-A were granted by funding from the Community of Madrid in the framework of the aforementioned FOODAL project. The CBGP was granted “Severo Ochoa” Distinctions of Excellence by the Spanish Ministry of Science and Innovation (SEV-2016-0672 and CEX2020-000999-S).

## Acknowledgments

The authors would like to thank ICTS-CNME (Universidad Complutense de Madrid, Madrid, Spain) for their help with the electronic microscopy assays, as well as Centro de Tecnología Biomédica – CTB (Universidad Politécnica de Madrid, Madrid, Spain) for their help with the DLS analyses.

## Conflict of interest

The authors declare that the research was conducted in the absence of any commercial or financial relationships that could be construed as a potential conflict of interest.

## Publisher’s note

All claims expressed in this article are solely those of the authors and do not necessarily represent those of their affiliated organizations, or those of the publisher, the editors and the reviewers. Any product that may be evaluated in this article, or claim that may be made by its manufacturer, is not guaranteed or endorsed by the publisher.

## Supplementary material

Supplementary Figure 1
*Nicotiana benthamiana* leaves agroinfiltrated with the different constructs. Leaves infiltrated with CP or Pru p 3-LF-CP (flexible linker) construct show clear bleaching patches, absent or almost absent in the Pru p 3-LH-CP (helicoidal linker) construct.
