# Corrigendum: Suitability of potyviral recombinant virus-like particles bearing a complete food allergen for immunotherapy vaccines

**DOI:** 10.3389/fimmu.2023.1133046

**Published:** 2023-01-19

**Authors:** Diego Pazos-Castro, Clémence Margain, Zulema Gonzalez-Klein, Marina Amores-Borge, Carmen Yuste-Calvo, Maria Garrido-Arandia, Lucía Zurita, Vanesa Esteban, Jaime Tome-Amat, Araceli Diaz-Perales, Fernando Ponz

**Affiliations:** ^1^ Centre for Plant Biotechnology and Genomics, Universidad Politécnica de Madrid – Instituto Nacional de Investigación y Tecnología Agraria y Alimentaria/Consejo Superior de Investigaciones Científicas (UPM–INIA/CSIC), Universidad Politécnica de Madrid, Madrid, Spain; ^2^ Department of Biotechnology-Plant Biology, Escuela Técnica Superior de Ingeniería Agronómica, Alimentaria y de Biosistemas (ETSIAAB), Universidad Politécnica de Madrid, Madrid, Spain; ^3^ Department of Allergy and Immunology, Instituto de Investigación Sanitaria (IIS)-Fundación Jiménez Díaz, Universidad Autónoma de Madrid (UAM), Madrid, Spain

**Keywords:** virus-like particles, antigen delivery, food allergy, immunotherapy, plant biotechnology, turnip mosaic virus, Pru p 3

In the published article, there was an error in the author list, and author Marina Amores-Borge was erroneously excluded. The corrected author list appears below.

“Diego Pazos-Castro, Clémence Margain, Zulema Gonzalez-Klein, Marina Amores-Borge, Carmen Yuste-Calvo, Maria Garrido-Arandia, Lucia Zurita, Vanesa Esteban, Jaime Tome-Amat, Araceli Diaz-Perales, Fernando Ponz”

In the published article, there was an error. The missing author was not included in the **Author Contributions** section.

A correction has been made to **Author contributions.** This sentence previously stated:

“DP-C: Conceptualization, investigation, writing original draft. CM: Investigation. ZG-K: Conceptualization, investigation, writing review. CY-C: Investigation. MG-A: Investigation, writing review. LZ: Investigation. VE: Writing review, resources, funding acquisition. JT-A: Conceptualization, investigation, writing original draft. AD-P: Conceptualization, writing original draft, resources, funding acquisition. FP: Conceptualization, writing review, resources, funding acquisition. All authors contributed to the article and approved the submitted version.”

The corrected sentence appears below:

“DP-C: Conceptualization, investigation, writing original draft. CM: Investigation. ZG-K: Conceptualization, investigation, writing review. MA-B: Investigation. CY-C: Investigation. MG-A: Investigation, writing review. LZ: Investigation. VE: Writing review, resources, funding acquisition. JT-A: Conceptualization, investigation, writing original draft. AD-P: Conceptualization, writing original draft, resources, funding acquisition. FP: Conceptualization, writing review, resources, funding acquisition. All authors contributed to the article and approved the submitted version.”

The authors apologize for these errors and state that they do not change the scientific conclusions of the article in any way. The original article has been updated.

