# Supplementary figures and images for: Suitability of potyviral recombinant virus-like particles bearing a complete food allergen for immunotherapy vaccines
Source: Front Immunol. 2022 Sep 8;13:986823. doi: 10.3389/fimmu.2022.986823 (PMC9492988; doi:10.3389/fimmu.2022.986823)

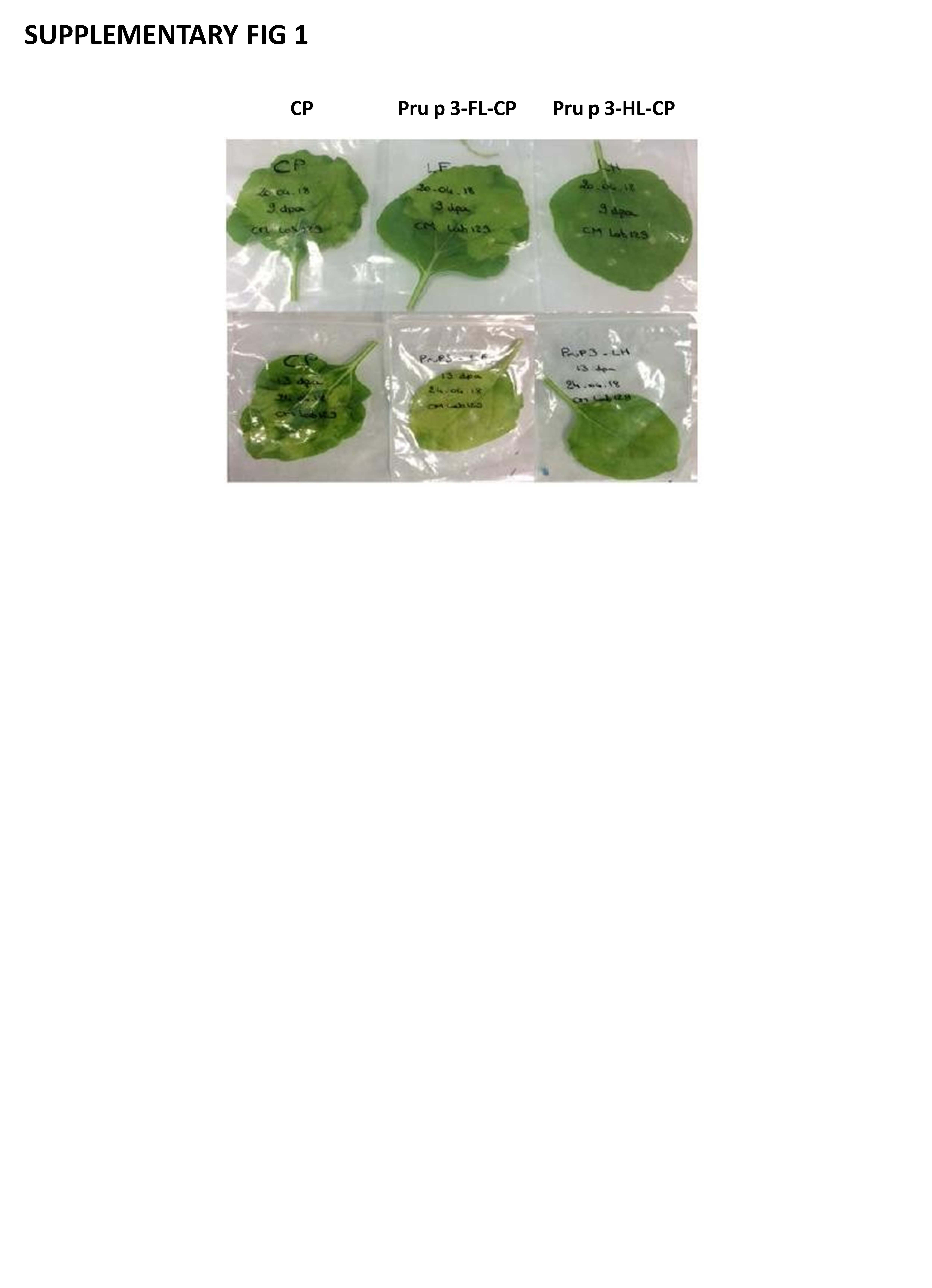

Supplement: Supplementary Figure 1 — Nicotiana benthamiana leaves agroinfiltrated with the different constructs. Leaves infiltrated with CP or Pru p 3-LF-CP (flexible linker) construct show clear bleaching patches, absent or almost absent in the Pru p 3-LH-CP (helicoidal linker) construct. [file Image_1.tif]
